# Standard toxicity study of clinical-grade allogeneic human bone marrow-derived clonal mesenchymal stromal cells

**DOI:** 10.1186/s13287-022-02899-9

**Published:** 2022-05-26

**Authors:** Behnoosh Tayebi, Mahnaz Babaahmadi, Mohammad Pakzad, Mostafa Hajinasrollah, Farhad Mostafaei, Shahrbanoo Jahangiri, Amir Kamali, Hossein Baharvand, Mohamadreza Baghaban Eslaminejad, Seyedeh-Nafiseh Hassani, Ensiyeh Hajizadeh-Saffar

**Affiliations:** 1 Department of Applied Cell Sciences, Faculty of Basic Sciences and Advanced Medical Technologies, Royan Institute, ACECR, Tehran, Iran; 2 Department of Stem Cells and Developmental Biology, Cell Science Research Center, Royan Institute for Stem Cell Biology and Technology, ACECR, Tehran, Iran; 3 Animal Core Facility, Reproductive Biomedicine Research Center, Royan Institute for Animal Biotechnology, ACECR, Tehran, Iran; 4 Advanced Therapy Medicinal Product Technology Development Center (ATMP-TDC), Royan Institute for Stem Cell Biology and Technology, ACECR, Tehran, Iran; 5 Labra Laboratory, Tehran, Iran; 6 Department of Developmental Biology, School of Basic Sciences and Advanced Technologies in Biology, University of Science and Culture, Tehran, Iran; 7 Department of Regenerative Medicine, Cell Science Research Center, Royan Institute for Stem Cell Biology and Technology, ACECR, Tehran, Iran

**Keywords:** Toxicity, Bone marrow, Clonal mesenchymal stromal cells, GMP, Tumorigenicity

## Abstract

**Introduction:**

Mesenchymal stromal cells (MSCs) have opened a new window to treat inflammatory and non-inflammatory diseases. Nonetheless, their clinical applications require rigorous control and monitoring procedures to ensure full compliance with the principles of good manufacturing practice (GMP). Various evaluations should be passed in conjunction with the development of these newly emerging therapeutic products from bench-to-bedside. These evaluations include in vitro characterization, preclinical studies, and clinical trials to ensure product safety and efficacy. Therefore, a robust and well-designed preclinical study is critical to confirm product safety. This study aims to determine the probable toxicity effects of local and systemic injections of cryopreserved human bone marrow-derived clonal MSCs (BM-cMSCs) during subacute and subchronic periods of time.

**Methods:**

BM-cMSCs were characterized according to the International Society for Cell and Gene Therapy (ISCT) criteria for MSCs. Both safety and toxicity of the BM-cMSCs population produced under GMP-compatible conditions were assessed in both sexes of Sprague Dawley (SD) rats via systemic intravenous (IV) administration and local injection in intervertebral disc (IVD). Behavioral changes, clinical signs of toxicity, and changes in body weight, water and food consumption were the important variables for product toxicity testing over 14 consecutive days during the subacute period and 90 consecutive days during the subchronic period. At the end of the assessment periods, the rats were killed for histopathology analysis of the target tissues. The BM-cMSCs potential for tumorigenicity was checked in nude mice.

**Results:**

Single IV and IVD injections of BM-cMSCs did not cause significant signs of clinical toxicity, or changes in laboratory and histopathology data during the subacute (14 day) and subchronic (90 day) periods. Ex vivo-expanded and cryopreserved BM-cMSCs did not induce tumor formation in nude mice.

**Conclusion:**

The results suggest that local and systemic administrations of xenogeneic BM-cMSCs in both sexes of SD rats do not cause toxicity during the subacute and subchronic periods of time. Also, BM-cMSCs were non-tumorigenic in nude mice.

**Supplementary Information:**

The online version contains supplementary material available at 10.1186/s13287-022-02899-9.

## Introduction

Bone marrow-derived mesenchymal stromal cells (BM-MSCs) have remarkable immunomodulation potential and trophic effects for treating various autoimmune and inflammatory diseases, and this has encouraged researchers and industries to use them as cell-based medicinal products [1, 2]. BM-MSCs can provide hope for treating diseases that lack definitive treatment, such as critical limb ischemia, acute myocardial ischemia, inflammatory bowel disease, chronic low back pain (cLBP), graft-versus-host disease (GvHD), osteoarthritis (OA), rheumatoid arthritis (RA), and multiple sclerosis (MS) [3–7]. Since MSCs lack major histocompatibility complex class II (MHC II) molecules on their surface, they have very low immunogenicity; therefore, they can be produced as “off-the-shelf” drugs for allogeneic transplantation applications [3, 8–10].

Despite the expanding use of MSCs (1140 trials; source: http://www.clinicaltrials.gov, "Mesenchymal Stem OR Mesenchymal Stromal” queried in September, 2021), only a few could pass from academic assessments to industrial platforms [1]. High costs of its commercialization are mainly attributed to current challenging regulatory requirements that must be met through intricate production procedures which require specific clean rooms with certain specifications, equipment and infrastructure, and also massive quality control tests and comprehensive validation protocols [11]. Scale-up and production of a heterogeneous population of various progenitor cell types that are committed to mesoderm lineages obtained from the isolation of BM-MSCs by conventional methods impedes their entry into the industry [12]. The inherent heterogeneity of BM-MSCs and their effects on cell culture, including premature aging of the cell culture that precludes mass cultivation and the discrepancy in inflammatory responses that lead to mixed, inconsistent in vivo results are disadvantages of these cells [13, 14].

The development of bone marrow-derived clonal MSCs (BM-cMSCs) provides a potential method for producing homogenous MSCs. The creation of an allogeneic bank of BM-cMSCs from distinct colonies by subfractionation culturing method [15, 16] from BM samples of healthy and young volunteers may overcome the previously mentioned complications [13–15, 17, 18]. BM-cMSCs have the same criteria as other sources of MSCs, such as the expression of specific MSC-related markers. Therefore, these cells can be promising choices for MSC-based therapeutic applications [12].

Safety is one of the critical features for clinical use of a cell-based product; in addition to in vitro studies, robust evaluations in preclinical studies are needed to confirm both safety and non-toxicity of the product [2, 19].

According to the existing guidelines, ICH: S6(R1) [20] and EMEA/CHMP/410869/2006 [21], a preclinical study’s objective is to elucidate the safety of the proposed protocol. This objective includes defining the biological component, its toxicity, and the response or reaction that the human body may manifest. This response should be identified before each clinical trial and during all clinical trial phases (ICHS6: ICHS6 (R1) [20, 22]. Preclinical studies can provide important information about safe doses for clinical trials, the treatment protocol, duration of exposure, time required for diagnosing side effects, identification of target organs in terms of toxicity and controlling parameters, and analysis of expected risk factors [21, 23–26].

Preclinical assessments must be undertaken to develop clinical-grade BM-cMSCs as an advanced therapy medicinal product (ATMP). Here, we performed preclinical analyses of the final good manufacturing practice (GMP)-compatible pharmaceutical product to address general toxicity and tumorigenicity of these cryopreserved BM-cMSCs. Both subacute and subchronic toxicities of intravenous (IV) and intervertebral disc (IVD) administrations of BM-cMSCs in both sexes of Sprague Dawley (SD) rats were assessed, and tumorigenicity in nude mice.

## Materials and methods

### Animal care

All experimental animal procedures were performed in accordance with the standard guidelines of the NIH Guide for the Care and Use of Laboratory Animals (8th edition) [27].

A total of 96 adult SD rats of both sexes were supplied from the Animal Core Facility of Royan Institute, Tehran, Iran. The animals were kept in polypropylene cages in a controlled room at 18–24 °C with a 12-h light/dark cycle, relative humidity of 30%–70%, and free access to food and water.

### Production and characterization of bone marrow-derived clonal mesenchymal stromal cells (BM-cMSCs)

In this study, BM-cMSCs were supplied by the Royan ATMP Technology Development Center (Royan ATMP-TDC). Establishment of the human BM-cMSC cell bank was previously described [16]. Briefly, BM was aspirated from the iliac crest of a healthy donor after receipt of written informed consent. A total of 2 ml BM was transferred to a 100-mm tissue culture dish (Corning, 430,167, USA) that Minimum Essential Medium Eagle-Alpha Modification (Alpha MEM; Thermo Fisher Scientific, 11,900,024, USA) supplemented with 20% fetal bovine serum (FBS; HyClone, SH30070-03, USA). The tissue culture dish was incubated at 37 °C and 5% CO_2_. The supernatant that contained the desired cells was transferred to new culture dishes on days (D) 2, 3, 4, and 5 and incubated until approximately D21. The dishes were marked as D2 to D5. Then, the desired single colonies in the dishes were separated and passaged using cloning cylinders (GIBCO-BRL) and treated with TrypLE enzyme (Thermo Fisher, A12177-03, USA) into six-well plates (Corning, 3516, USA). In the successive serial passages, the cells were passaged every 5 to 6 days and placed in the appropriate vessels. The cells were fully characterized and banked in a four-tiered cell bank comprising the following tiers: initial (ICB), master (MCB), working (WCB), and end of product (EoPCB). Cells from all cell banks were characterized according to minimal criteria set by the International Society for Cell and Gene Therapy (ISCT) for MSCs [16, 28], and the certificate of analysis of these cells has been documented in the correlating article [16]. Briefly, the analysis showed high expression (> 95%) of MSC markers (CD90, CD105, CD73) and decreased expression (< 2%) of hematopoietic markers (CD34, CD45, CD14, CD79α) with the human MSC Phenotyping Kit (MACS Miltenyi Biotec, 130-095-198, USA). Passage-15 cells were examined for viability (> 90%) and karyotyping, and there were no observed genomic abnormalities. Microbial and mycoplasma contamination were not seen, and the endotoxin level was within the permissible limits. These cMSCs could undergo multi-lineage differentiation, which was confirmed by adipogenic, chondrogenic, and osteogenic differentiation kits (Thermo Fisher, A1007001, USA). All characterizations were published in [16].

### In vivo transplantation of bone marrow-derived clonal mesenchymal stromal cells (BM-cMSCs)

**Fig. 1:**
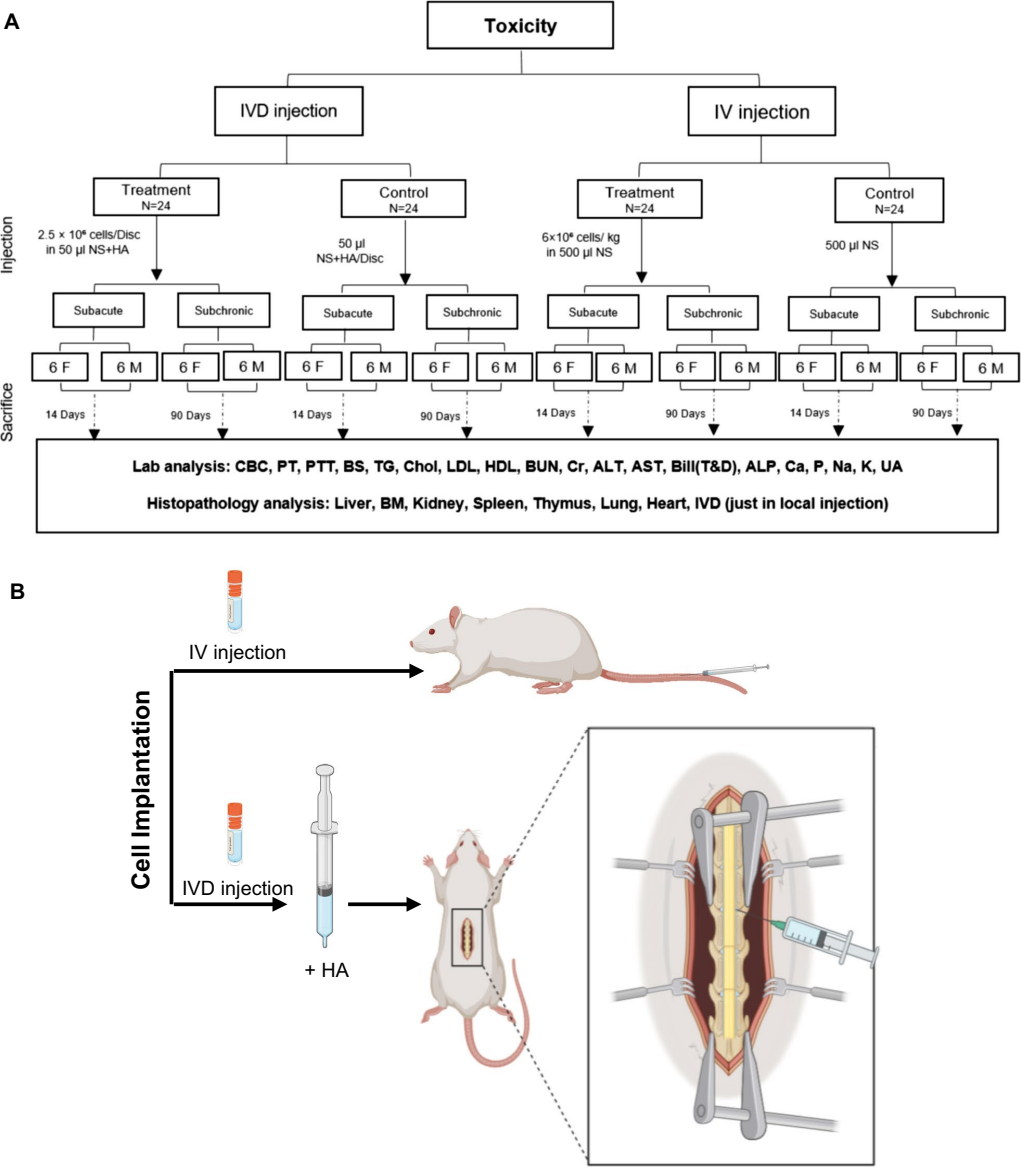
The overall program for injection of BM-cMSCs. **A** Injections were performed by two different routes in the treatment and control groups. In the IVD injection route, 2.5 × 10^6^ cells were suspended in NS mixed with an equal volume of HA. In the IV injection route, 6 × 10^6^ cells/kg body weight in 500 μl NS were injected in the treatment group and 500 μl NS in the control group. The animals were followed for 14 days (subacute) and 90 days (subchronic). At the end of the subacute and subchronic phases, the animals were euthanized and their tissues were processed for hematological and biochemistry laboratory assessments, and histopathology analysis. For each administration route, we used 48 Sprague Dawley (SD) rats of both sexes (*n* = 24 female; *n* = 24 male). **B** The schematic of surgery and method of cell injection into the disc is shown. For IV injection route, cells were injected in the tail vein. For IVD injection route, laminectomy was performed on the dorsal region to expose the lumbar spine. The injection was performed on disc located between the L4 and L5 vertebrae by a 21G needle. After injection, the surgical wounds were closed routinely. BM-cMSCs: bone marrow-derived clonal MSCs; IVD: intervertebral disc; NS: normal saline; HA: hyaluronic acid; IV: intravenous

The male and female rats were randomly assigned to two main injection groups—IV and local injection into IVD. Subacute (14 days) [29] and subchronic (90 days) [29] toxicity parameters were examined following the injections. Figure 1A shows the overall toxicity study design.

It is shown that the amount of 1.8 × 10^7^ cells per disc were injected in a cLBP clinical trial [30] and 2 × 10^6^ cells/kg body weight in an RA study [31]. In the current study, for the IVD group, we took into consideration the differences in disc surface area between humans (1727 mm^2^) [32] and rats (20.4 mm^2^) [32] and determined that 0.25 × 10^6^ cells should be injected per disc for the rats (Table 1). In order to determine the highest injectable dose, we multiplied the previously defined dose by 10 to obtain 2.5 × 10^6^ cells. Cryopreserved BM-cMSCs were thawed in a 37 °C water bath. Cell counting was done both manually using Neubauer hemocytometer (trypan blue) and automatically by NucleoCounter®NC-200™ (ChemoMetec, Denmark). Our validation study showed that the cell viability was more than 90% after thawing. The cells rinsed once with normal saline (NS) through centrifugation and re-suspended in 25 μl of NS with an equal volume of hyaluronic acid (HA; HYAULBRIX, Fidia, 1.5%) as the carrier for the injection. Next, the rats were placed under general anesthesia by IV administration of 80 mg/kg ketamine (Alfasan, Holland) and 5 mg/kg xylazine (Alfasan, Holland). A laminectomy was performed to open the dorsal region and expose the lumbar spine of each rat. The cMSC-HA combination was injected into L4–L5 intervertebral disc (as the most common disc injury in humans) by 21G needle. After injection, the surgical wounds were closed routinely (Fig. 1B). The animals were monitored for postoperative complications of bleeding, infection, ulcers, spinal cord injury, and ruptured abdominal blood vessels. Enrofloxacin 10% (Aburaihan Pharma, Iran) 5 mg/kg and tramadol (Alborz Darou, Iran) 15 mg/kg were injected subcutaneously for 3 days to control infection and pain, respectively.

**Table 1:** Comparison of lumbar disc surface and height in humans and rats

Species	Disc surface (mm^2^)	Disc height (mm^2^)
Human	1727	8.93
Rat (lumbar disc)	20.4	0.93

Animals in the IV group received the cMSCs via their tail veins. We used the highest possible dose, which was three times more than the common clinical trial dose of 6 × 10^6^ cells/kg [31]. The cMSCs were dissolved in a carrier solution of 500 μl NS prior to administration.

In addition, carrier was given to the two control groups (equal combination of NS and HA for IVD group and NS for IV group).

### Subacute and subchronic toxicity assessments

For this study, from a total of 96 rats, 48 were selected for each group (IV and IVD). In each group, rats were divided into two subgroups of subacute and subchronic periods which were then further divided into treatment and control groups. In each of these groups, 12 rats were assigned (six males and six females) (Fig. 1A). Animals were monitored for the following signs of subacute (14 days) and subchronic (90 days) toxicities: behavioral and clinical symptoms, water and food consumption, and mortality (Additional file 1: Table S1). For subacute toxicity evaluation, on day 15, six male and six female rats from each group were anesthetized by intraperitoneal administration of 80 mg/kg ketamine and 10 mg/kg xylazine and then euthanized with carbon dioxide in order to perform the necessary laboratory evaluations (hematological parameters, biochemistry, coagulation tests, and urinalysis).

Moreover, as for subchronic toxicity evaluation, subjects were examined twice weekly for 90 days to assess behavioral and clinical symptoms of toxicity, water and food consumption, and mortality. On day 90, the rats were anesthetized and then euthanized with carbon dioxide in order to perform the necessary laboratory tests (hematological and biochemistry parameters, coagulation tests, and urinalysis) and additional pathology investigations in selected tissues.

### Histopathology analysis

After the animals were euthanized, the harvested tissues (heart, kidneys, liver, spleen, thymus, and lungs) were fixed in 10% neutral buffered formalin (NBF, pH 7.26) for 48 h, then processed, embedded in paraffin, and sectioned into 5-μm-thick sections. After staining with hematoxylin and eosin (H&E), two independent pathologists evaluated the slides under a light microscope (Olympus BX51; Olympus, Tokyo, Japan). Acute and chronic inflammatory response, fatty changes, coagulative necrosis, hemorrhage, hyperemia, and any changes with regards to the normal tissue structure were assessed in the different samples.

### In vivo tumorigenicity assay

Nude mice (six weeks old, male) were obtained from the Animal Core Facility at Royan Institute (Tehran, Iran) 2 weeks before the cell injections. The animals were maintained in a clean room in individually ventilated cages. The treatment group received 5 × 10^6^ cells mixed with 50 μl ice cold Matrigel (1:1, Sigma-Aldrich), and the negative control group received 100 μl Matrigel via intradermal injections in three different areas. The Royan H6 human embryonic stem cell (hESC) line (Royan Stem Cell Bank) was the positive control. All mice were examined, and tumor measurements were recorded twice weekly for 3 months. After anesthetizing by intraperitoneal administration of 80 mg/kg ketamine and 8 mg/kg xylazine, the animals were sacrificed by inhaling carbon dioxide, and the histopathological assessments were performed at the injection sites for teratoma formation.

### Statistical analysis

Statistical analysis for the changes in body weight, water and food consumption, hematological parameters, and biochemical assessments was performed using IBM SPSS version 25 (IBM Corp., Armonk, NY, USA) and Prism 8 (GraphPad Software., La Jolla California, USA) software. The results were recorded as mean ± SD. Significant differences between the control and treatment groups were assessed by the independent samples *t* test, two-way repeated measures ANOVA, and multiple comparisons. Two-way repeated measures ANOVA and multiple comparisons were performed to compare the changes in the two groups over the period of the time. *P* values less than 0.05 were considered statistically significant. The graphs were plotted with GraphPad Prism 8 software.

## Results

### Single injection of BM-cMSCs via IVD and IV administration did not cause toxicity during subacute and subchronic periods

We did not observe any toxicity-related abnormal clinical signs or cell-related mortality in the rats during the subacute and subchronic assessment times. However, one animal from the IVD group died during surgery and one from the IV control group was euthanized after 27 days (Tables 2 and 3) due to severe clinical symptoms from an external parasite. All of the other animals had normal behavioral changes and normal clinical signs during this period.

**Table 2:** Percentage of deaths in IVD-studied groups in subacute and subchronic phase

Groups	Number of animals per group		Dose levels	Mortality (%)	
	Subacute phase	Subchronic phase		Subacute phase	Subchronic phase
Control	12 (6 males and 6 females)	12 (6 males and 6 females)	50 μl (NS + HA)/disc	0	0
Treatment	12 (6 males and 6 females)	12 (6 males and 6 females)	2.5 × 10^6^ cells/disc in 50 μl (HA + NS)	8.3 (one female)	0

*IVD* intervertebral disc; *NS* normal saline; *HA* hyaluronic acid

**Table 3:** Percentage of deaths in IV-studied groups in subacute and subchronic phase

Groups	Number of animals per group		Dose levels	Mortality (%)	
	Subacute phase	Subchronic phase		Subacute phase	Subchronic phase
Control	12 (6 males and 6 females)	12 (6 males and 6 females)	500 μl NS	0	8.3 (one male)
Treatment	12 (6 males and 6 females)	12 (6 males and 6 females)	6 × 10^6^ cells/kg in 500 μl NS + 1 μl Rock inhibitor	0	0

*IV* intravenous, *NS* normal saline

**Fig. 2:**
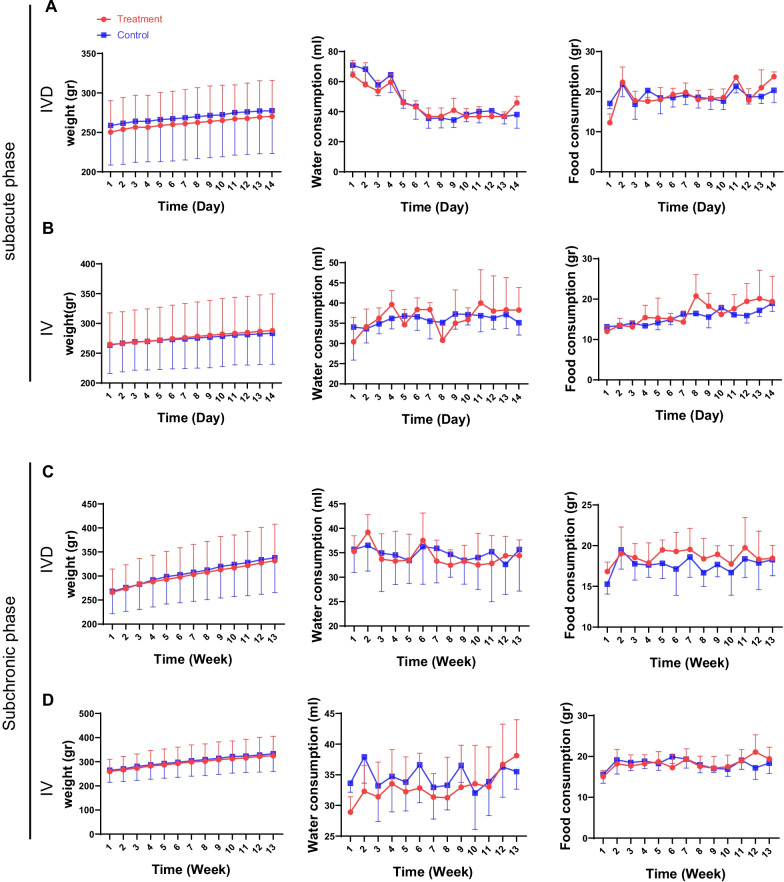
Weight, water, and food consumption of the rats. Weight, water, and food consumption comparison chart in treatment and control groups in subacute toxicity study; IVD injection (**A**), IV injection (**B**) and in subchronic toxicity study; IVD injection (**C**), IV injection (**D**) is shown; P value ≤ 0.05

According to the results, the IV and IVD cell injections had no effects on the weight of the rats during the subacute and subchronic periods. A gradual increase in body weight of all the animals was observed (Fig. 2). The total body weight of animals at the end of the 14th and 90th days was not statistically significant compared to the control group (Additional file 1: Tables S2 and S3). Absolute water (Additional file 1: Tables S4 and S5) and food consumption (Additional file 1: Tables S6 and S7) were not statistically significant in comparison with the control group (Fig. 2). Although changes in food consumption in the IVD group during the subacute period at D1, D4, and D11 were statistically significant, as well as water consumption in the IV group during the subchronic period at W1, these changes were transient and minimal.

Hematological, biochemical, and coagulation evaluations showed no significant differences in any laboratory parameters between the control and treatment groups. In the IVD group, the hemoglobin and hematocrit levels of the treatment and control groups were lower than the normal range in the subacute phase (Tables 4, 5, 6, and 7).

**Table 4:** Pooled hematological parameters (male and female) in the IVD groups for the study of subacute and subchronic toxicities of human BM-cMSCs

Parameters	Subacute phase			Subchronic phase		
	Treatment group	Control	*P* value	Treatment group	Control	*p* value
Number of animals analyzed	11	12		12	12	
WBCs (× 10^3^/μl)	11.17 ± 3.46	11.07 ± 1.79	0.93	8.26 ± 1.26	8.26 ± 1.26	0.51
RBCs (× 10^6^/μl)	5.91 ± 0.71	6.33 ± 0.69	0.17	6.42 ± .40	6.42 ± 0.40	0.22
HGB (g/dl)	11.08 ± 0.95	11.60 ± 1.07	0.23	12.31 ± .84	12.31 ± 0.84	1.0
HCT (%)	31.10 ± 3.05	31.46 ± 3.50	0.79	32.67 ± 2.03	32.67 ± 2.03	0.97
MCV (fl)	50.51 ± 1.91	50.20 ± 2.51	0.74	49.74 ± 1.14	49.74 ± 1.14	0.86
MCH (pg)	18.79 ± 1.54	17.60 ± 1.49	0.07	19.61 ± .85	19.61 ± .85	1.0
MCHC (g/dl)	36.64 ± 0.97	36.42 ± 0.97	0.59	38.90 ± 1.23	38.90 ± 1.23	0.90
PLT (10^3^/μl)	485.74 ± 60.97	505.42 ± 45.0	0.38	542.08 ± 70.93	542.08 ± 70.93	0.59
RDW (%)	12.51 ± 1.24	12.35 ± 1.17	0.75	11.31 ± .53	11.31 ± 0.53	0.67
Reticulocytes (%)	1.86 ± 0.28	1.85 ± 0.21	0.25	1.08 ± 0.16	1.08 ± 0.16	0.91

*IVD* intervertebral disc; *BM-cMSC* bone marrow-derived clonal mesenchymal stromal cells; *WBCs* white blood cells; *RBCs* red blood cells; *HGB* hemoglobin; *HCT* hematocrit; *MCV* mean corpuscular volume; *MCH* mean corpuscular hemoglobin; *MCHC* mean corpuscular hemoglobin concentration; *PLT* platelets; *RDW-CV* red blood cell distribution width coefficient variation. *P* ≤ 0.05 indicates statistical significance

**Table 5:** Pooled hematological parameters (male and female) in the IV groups for the study of subacute and subchronic toxicities of human BM-cMSCs

Parameters	Subacute phase			Subchronic phase		
	Treatment group	Control	*P* value	Treatment group	Control	*P* value
Number of animals analyzed	12	12		12	11	
WBCs (× 10^3^/μl)	8.55 ± 2.12	8.23 ± 1.65	0.68	9.0 ± 1.96	8.03 ± 1.33	0.18
RBCs (× 10^6^/μl)	6.50 ± 0.68	6.31 ± 0.62	0.49	6.41 ± 0.51	6.21 ± 0.53	0.38
HGB (g/dl)	12.56 ± 0.37	12.46 ± 0.74	0.68	12.52 ± 0.76	12.18 ± 0.50	0.22
HCT (%)	36.56 ± 1.05	34.62 ± 4.73	0.19	33.25 ± 2.48	32.63 ± 2.81	0.58
MCV (fl)	52.72 ± 4.03	52.18 ± 3.24	0.72	50.62 ± 1.77	50.24 ± 1.60	0.59
MCH (pg)	20.52 ± 1.28	20.12 ± 1.98	0.56	19.71 ± 1.02	19.78 ± 1.29	0.89
MCHC (g/dl)	37.15 ± 2.64	37.04 ± 2.36	0.91	38.94 ± 1.88	39.20 ± 1.68	0.73
PLT (10^3^/μl)	472.16 ± 29.44	471.31 ± 43.55	0.95	531.50 ± 125.49	545.0 ± 110.63	0.78
RDW (%)	12.45 ± 1.01	12.65 ± 1.34	0.67	11.33 ± 0.92	11.03 ± 0.82	0.42
Reticulocytes (%)	0.97 ± 0.43	1.04 ± 0.54	0.74	1.10 ± 0.22	1.04 ± 0.03	0.39

*IV* intravenous; *BM-cMSC* bone marrow-derived mesenchymal stromal cells; *WBCs* white blood cells; *RBCs* red blood cells; *HGB* hemoglobin; *HCT* hematocrit; *MCV* mean corpuscular volume; *MCH* mean corpuscular hemoglobin; *MCHC* mean corpuscular hemoglobin concentration; *PLT* platelets; *RDW-CV* red blood cell distribution width coefficient variation. *P* ≤ 0.05 indicates statistical significance

**Table 6:** Pooled biochemical parameters (male and female) in the IVD groups for the study of subacute and subchronic toxicities of human BM-cMSCs

Parameters	Subacute phase			Subchronic phase		
	Treatment group	Control	*P* value	Treatment group	Control	*P* value
Number of animals analyzed	11	12		12	12	
Glucose (mg/dl)	144.50 ± 9.20	146.70 ± 13.80	0.66	118.5 ± 7.42	121.66 ± 6.82	0.3
Urea (mg/dl)	61.00 ± 6.94	57.16 ± 3.92	0.11	45.66 ± 4.11	46.0 ± 3.41	0.83
Cr (mg/dl)	0.60 ± 0.11	0.55 ± 0.12	0.34	0.68 ± 0.10	0.69 ± 0.12	0.77
ALT (U/l)	57.90 ± 12.08	59.25 ± 10.81	0.78	55.16 ± 6.13	55.08 ± 7.46	0.97
AST (U/l)	131.36 ± 42.01	138.58 ± 26.89	0.62	113.83 ± 11.59	119.41 ± 12.22	0.26
ALP (U/l)	769.54 ± 106.95	763.08 ± 91.82	0.87	303.91 ± 68.75	319.50 ± 60.06	0.56
Chol (mg/dl)	54.54 ± 7.78	60.83 ± 10.60	0.12	49.50 ± 9.86	48.58 ± 11.37	0.83
TG (mg/dl)	71.27 ± 23.27	71.16 ± 20.19	0.99	61.33 ± 13.95	58.00 ± 15.59	0.58
TP (g/dl)	5.57 ± 0.58	5.44 ± 0.69	0.63	6.39 ± 0.82	6.26 ± .43	0.64
Alb (g/dl)	3.08 ± 0.341	3.18 ± 0.30	0.5	3.12 ± 0.50	3.03 ± .40	0.63
TB (mg/dl)	0.15 ± 0.02	0.15 ± 0.03	0.58	0.22 ± 0.05	0.21 ± 0.05	0.75
DB (mg/dl)	0.08 ± 0.03	0.07 ± 0.02	0.53	0.12 ± 0.02	0.14 ± 0.02	0.09
P (mg/dl)	6.47 ± 1.08	6.41 ± 1.08	0.89	4.97 ± 0.61	5.05 ± .62	0.77
Ca (mg/dl)	8.50 ± 0.36	8.30 ± 0.40	0.25	7.85 ± 0.47	8.00 ± .54	0.50
Na (mEq/l)	140.9 ± 2.62	140.75 ± 3.16	0.89	138.50 ± 2.23	139.41 ± 3.17	0.42
K (mEq/l)	4.67 ± 0.82	5.23 ± 0.81	0.11	4.00 ± 0.28	4.00 ± 0.28	0.94
Cl (mEq/l)	108.45 ± 4.88	105.50 ± 4.31	0.13	103.58 ± 2.23	104.66 ± 1.87	0.21
HDL (mg/dl)	40.54 ± 2.62	41.41 ± 2.35	0.41	43.50 ± 2.06	42.91 ± 2.42	0.53
LDL (mg/dl)	33.36 ± 3.29	32.91 ± 3.47	0.75	34.50 ± 2.46	34.25 ± 2.66	0.81
PT (sec)	17.54 ± 1.34	17.70 ± 1.50	0.79	18.09 ± 0.88	18.45 ± 0.91	0.33
PTT (sec)	37.80 ± 4.97	36.32 ± 5.15	0.49	34.20 ± 3.55	34.47 ± 3.23	0.84

*BM-cMSC* bone marrow-derived mesenchymal stromal cells;* IVD* intervertebral disc; *Cr* creatinine; *ALT* alanine aminotransferase; *AST* aspartate transaminase; *ALP* alkaline phosphatase; *Chol* cholesterol; *TG* triglycerides; *TP* total protein; *Alb* albumin; *TB* total bilirubin; *DB* direct bilirubin; *P* phosphorus; *Ca* calcium; *Na* sodium; *K* potassium; *CL* chloride; *HDL* high-density lipoprotein cholesterol; *LDL* low-density lipoprotein cholesterol; *SD* standard deviation. *P* ≤ 0.05 indicates statistical significance

**Table 7:** Pooled biochemical parameters (male and female) in the IV groups for the study of subacute and subchronic toxicities of human BM-cMSCs

Parameters	Subacute phase			Subchronic phase		
	Treatment group	Control	*P* value	Treatment group	Control	*P* value
Number of animals analyzed	12	12		12	11	
Glucose (mg/dl)	145.91 ± 7.94	151.43 ± 11.68	0.19	134.33 ± 11.93	128.81 ± 13.36	0.30
Urea (mg/dl)	54.08 ± 4.96	51.08 ± 4.87	0.14	48.00 ± 5.57	49.27 ± 4.45	0.55
Cr (mg/dl)	0.65 ± 0.10	0.60 ± 0.10	0.35	0.70 ± 0.09	0.76 ± 0.11	0.15
ALT (U/l)	49.25 ± 5.18	51.08 ± 6.50	0.45	56.58 ± 6.25	55.09 ± 6.26	0.57
AST (U/l)	103.50 ± 9.11	102.91 ± 10.76	0.88	101.66 ± 8.65	102.36 ± 7.07	0.83
ALP (U/l)	519.08 ± 174.52	584.44 ± 149.31	0.33	365.91 ± 109.15	324.0 ± 75.54	0.30
Chol (mg/dl)	54.16 ± 7.35	53.75 ± 7.74	0.89	51.33 ± 9.80	51.09 ± 9.54	0.95
TG (mg/dl)	49.91 ± 17.35	54.83 ± 20.89	0.53	70.58 ± 19.03	64.81 ± 17.85	0.46
TP (g/dl)	6.01 ± .59	6.08 ± .74	0.81	6.21 ± .59	6.36 ± .66	0.57
Alb (g/dl)	3.42 ± .24	3.40 ± .21	0.83	3.37 ± .26	3.38 ± .37	0.96
TB (mg/dl)	0.76 ± 0.22	0.62 ± 0.18	0.09	0.18 ± 0.02	0.18 ± 0.03	0.65
DB (mg/dl)	0.15 ± 0.05	0.13 ± 0.04	0.26	0.10 ± 0.02	0.11 ± 0.02	0.50
P (mg/dl)	6.58 ± 0.79	7.19 ± 0.97	0.10	5.94 ± 0.29	5.99 ± 0.20	0.65
Ca (mg/dl)	8.35 ± 0.17	8.23 ± 0.31	0.27	8.09 ± 1.03	8.04 ± 0.51	0.89
Na (mEq/l)	140.66 ± 2.05	140.50 ± 2.11	0.84	139.16 ± 2.16	139.45 ± 1.80	0.73
K (mEq/l)	3.91 ± 0.22	4.05 ± 0.56	0.46	3.90 ± 0.27	3.65 ± 0.44	0.12
Cl (mEq/l)	107.66 ± 4.79	106.91 ± 5.35	0.72	103.83 ± 3.06	103.27 ± 3.37	0.68
HDL (mg/dl)	41.25 ± 2.83	41.08 ± 3.08	0.89	42.33 ± 2.96	41.54 ± 3.11	0.54
LDL (mg/dl)	33.00 ± 3.46	33.91 ± 2.93	0.49	33.00 ± 3.33	31.81 ± 2.78	0.36
PT (s)	17.97 ± 1.40	17.81 ± 0.90	0.74	17.95 ± 0.99	17.94 ± 0.96	0.97
PTT (s)	36.80 ± 2.76	35.97 ± 4.46	0.59	33.94 ± 2.79	33.32 ± 3.12	0.62

*BM-cMSC* bone marrow-derived mesenchymal stromal cells; *IV* intravenous; *Cr* creatinine; *ALT* alanine aminotransferase; *AST* aspartate transaminase; *ALP* alkaline phosphatase; *Chol* cholesterol; *TG* triglycerides; *TP* total protein; *Alb* albumin; *TB* total bilirubin; *DB* direct bilirubin; *P* phosphorus; *Ca* calcium; *Na* sodium; *K* potassium; *CL* chloride; *HDL* high-density lipoprotein cholesterol; *LDL* low-density lipoprotein cholesterol; *SD* standard deviation. *P* ≤ 0.05 indicates statistical significance

Macroscopic urinalysis was performed directly by using urine strips to determine pathological changes in each group. The tests were carried out within 60 s after soaking. The analysis included testing for the presence of hemoglobin, proteins, glucose, ketones, bilirubin, urobilinogen, acetone, nitrite, and leucocytes, and pH specific gravity. No significant clinical differences were found between the study groups (Tables 8 and 9).

**Table 8:** Results of macroscopic urinalysis in IVD groups for the study of subacute and subchronic toxicities

Groups	Parameters										
	Blood (RBC/μl)	Urobilinogen (mg/dl)	Bilirubin	Protein (mg/dl)	Nitrite	Ketone	Ascorbic acid	Glucose (mg/dl)	pH	SG	WBCs
*Subacute phase of cell injection*											
Treatment (female)	Neg	Neg	Neg	Neg	Neg	Neg	Neg	Neg	8	1015	Neg
	Neg	Neg	Neg	Neg	Neg	Neg	Neg	Neg	7	1020	Neg
	Trace	Neg	Neg	Neg	Neg	Neg	Neg	Neg	7	1015	Neg
	Neg	Neg	Neg	Neg	Neg	Neg	Neg	Neg	6	1020	Neg
	Neg	Neg	Neg	Neg	Neg	Neg	Neg	Neg	7	1015	Neg
Treatment (male)	Neg	Neg	Trace	Neg	Neg	Neg	Neg	Neg	6	1020	Neg
	Neg	Neg	Neg	Neg	Neg	Neg	Neg	Neg	7	1025	Neg
	Neg	Neg	Neg	Trace	Neg	Neg	Neg	Neg	7	1025	Neg
	Neg	Neg	Neg	Neg	Neg	Neg	Neg	Neg	6	1020	Neg
	Neg	Neg	Neg	Neg	Neg	Neg	Neg	Neg	6	1015	Neg
	Neg	Neg	Neg	Neg	Neg	Neg	Neg	Neg	7	1020	Neg
Control (female)	Neg	Neg	Neg	Neg	Neg	Neg	Neg	Neg	6	1030	Neg
	Neg	Neg	Neg	Neg	Neg	Neg	Neg	Neg	7	1020	Neg
	Trace	Neg	Neg	Neg	Neg	Neg	Neg	Neg	6	1010	Neg
	Neg	Neg	Neg	Neg	Neg	Neg	Neg	Neg	7	1015	Neg
	Neg	Neg	Neg	Trace	Neg	Neg	Neg	Neg	6	1015	Neg
	Neg	Neg	Neg	Neg	Neg	Neg	Neg	Neg	6	1020	Neg
Control (male)	Neg	Neg	Neg	Neg	Neg	Neg	Neg	Neg	7	1015	Neg
	Neg	Neg	Neg	Neg	Neg	Neg	Neg	Neg	7	1020	Neg
	Neg	Neg	Neg	Neg	Neg	Neg	Neg	Neg	6	1015	Neg
	Neg	Neg	Neg	Neg	Neg	Neg	Neg	Neg	6	1020	Neg
	Trace	Neg	Neg	Neg	Neg	Neg	Neg	Neg	6	1015	Neg
	Neg	Neg	Neg	Neg	Neg	Neg	Neg	Neg	7	1025	Neg
*Subchronic phase of cell injection*											
Treatment (female)	Neg	Neg	Neg	Neg	Neg	Neg	Neg	Neg	6	1020	Neg
	Neg	Neg	Neg	Neg	Neg	Neg	Neg	Neg	7	1030	Neg
	Neg	Neg	Neg	Neg	Neg	Neg	Neg	Neg	8	1015	Neg
	Neg	Neg	Neg	Neg	Neg	Neg	Neg	Neg	6	1015	Neg
	Neg	Neg	Neg	Neg	Neg	Neg	Neg	Neg	6	1020	Neg
	Neg	Neg	Neg	Neg	Neg	Neg	Neg	Neg	7	1015	Neg
Treatment (male)	Neg	Neg	Neg	Neg	Neg	Neg	Neg	Neg	6	1010	Neg
	Neg	Neg	Neg	Neg	Neg	Neg	Neg	Neg	7	1030	Neg
	Neg	Neg	Neg	Neg	Neg	Neg	Neg	Neg	7	1020	Neg
	Neg	Neg	Neg	Neg	Neg	Neg	Neg	Neg	6	1015	Neg
	Neg	Neg	Neg	Neg	Neg	Neg	Neg	Neg	6	1015	Neg
	Neg	Neg	Neg	Neg	Neg	Neg	Neg	Neg	7	1020	Neg
Control (female)	Neg	Neg	Neg	Neg	Neg	Neg	Neg	Neg	6	1010	Neg
	Neg	Neg	Neg	Neg	Neg	Neg	Neg	Neg	7	1020	Neg
	Neg	Neg	Neg	Neg	Neg	Neg	Neg	Neg	8	1015	Neg
	Neg	Neg	Neg	Neg	Neg	Neg	Neg	Neg	6	1020	Neg
	Neg	Neg	Neg	Neg	Neg	Neg	Neg	Neg	6	1015	Neg
	Neg	Neg	Neg	Neg	Neg	Neg	Neg	Neg	6	1015	Neg
Control (male)	Neg	Neg	Neg	Neg	Neg	Neg	Neg	Neg	7	1015	Neg
	Neg	Neg	Neg	Neg	Neg	Neg	Neg	Trace	7	1020	Neg
	Neg	Neg	Neg	Neg	Neg	Neg	Neg	Neg	6	1015	Neg
	Neg	Neg	Neg	Neg	Neg	Neg	Neg	Neg	7	1015	Neg
	Neg	Neg	Neg	Neg	Neg	Neg	Neg	Neg	7	1015	Neg
	Neg	Neg	Neg	Neg	Neg	Neg	Neg	Neg	6	1015	Neg

*IVD* intervertebral disc; *SG* specific gravity; *Neg* negative; *Pos* positive

**Table 9:** Results of macroscopic urinalysis in IV groups for the study of subacute and subchronic toxicities

Groups	Parameters										
	Blood (RBC/μl)	Urobilinogen (mg/dl)	Bilirubin	Protein (mg/dl)	Nitrite	Ketone	Ascorbic acid	Glucose (mg/dl)	pH	SG	WBCs
*Subacute phase of cell injection*											
Treatment (female)	Neg	Neg	Neg	Neg	Neg	Neg	Neg	Neg	6	1010	Neg
	Neg	Neg	Neg	Trace	Neg	Neg	Neg	Neg	7	1015	Neg
	Neg	Neg	Neg	Neg	Neg	Neg	Neg	Neg	6	1010	Neg
	Neg	Neg	Neg	Neg	Neg	Neg	Neg	Neg	7	1015	Neg
	Neg	Neg	Neg	Neg	Neg	Neg	Neg	Neg	7	1020	Neg
	Neg	Neg	Neg	Neg	Neg	Neg	Neg	Neg	6	1020	Neg
Treatment (male)	Neg	Neg	Neg	Neg	Neg	Neg	Neg	Neg	6	1015	Neg
	Neg	Neg	Neg	Neg	Neg	Neg	Neg	Neg	7	1025	Neg
	Neg	Neg	Neg	Neg	Neg	Neg	Neg	Neg	7	1020	Neg
	Neg	Neg	Neg	Neg	Neg	Neg	Neg	Neg	6	1015	Neg
	Neg	Neg	Neg	Neg	Neg	Neg	Neg	Neg	6	1015	Neg
	Neg	Neg	Neg	Neg	Neg	Neg	Neg	Neg	6	1015	Neg
Control (female)	Neg	Neg	Neg	Neg	Neg	Neg	Neg	Neg	6	1030	Neg
	Neg	Neg	Neg	Neg	Neg	Neg	Neg	Neg	6	1020	Neg
	Trace	Neg	Neg	Trace	Neg	Neg	Neg	Neg	6	1015	Neg
	Neg	Neg	Neg	Neg	Neg	Neg	Neg	Neg	7	1020	Neg
	Neg	Neg	Neg	Neg	Neg	Neg	Neg	Neg	6	1020	Neg
	Neg	Neg	Neg	Neg	Neg	Neg	Neg	Neg	7	1020	Neg
Control (male)	Neg	Neg	Neg	Neg	Neg	Neg	Neg	Neg	7	1015	Neg
	Neg	Neg	Neg	Neg	Neg	Neg	Neg	Neg	6	1020	Neg
	Neg	Neg	Neg	Neg	Neg	Neg	Neg	Neg	7	1010	Neg
	Neg	Neg	Neg	Neg	Neg	Neg	Neg	Neg	6	1015	Neg
	Neg	Neg	Neg	Neg	Neg	Neg	Neg	Neg	7	1020	Neg
	Neg	Neg	Neg	Neg	Neg	Neg	Neg	Neg	6	1020	Neg
*Subchronic phase of cell injection*											
Treatment (female)	Neg	Neg	Neg	Neg	Neg	Neg	Neg	Neg	8	1015	Neg
	Trace	Neg	Neg	Neg	Neg	Neg	Neg	Neg	8	1015	Neg
	Neg	Neg	Neg	Neg	Neg	Neg	Neg	Neg	7	1010	Neg
	Neg	Neg	Neg	Neg	Neg	Neg	Neg	Neg	6	1015	Neg
	Neg	Neg	Neg	Neg	Neg	Neg	Neg	Neg	6	1015	Neg
	Neg	Neg	Neg	Neg	Neg	Neg	Neg	Neg	6	1020	Neg
Treatment (male)	Neg	Neg	Neg	Neg	Neg	Neg	Neg	Neg	7	1015	Neg
	Neg	Neg	Neg	Neg	Neg	Neg	Neg	Neg	7	1015	Neg
	Neg	Neg	Neg	Neg	Neg	Neg	Neg	Neg	7	1010	Neg
	Neg	Neg	Neg	Neg	Neg	Neg	Neg	Neg	6	1015	Neg
	Neg	Neg	Neg	Neg	Neg	Neg	Neg	Neg	6	1015	Neg
	Neg	Neg	Neg	Neg	Neg	Neg	Neg	Neg	6	1020	Neg
Control (female)	Neg	Neg	Neg	Neg	Neg	Neg	Neg	Neg	7	1010	Neg
	Neg	Neg	Neg	Neg	Neg	Neg	Neg	Neg	8	1010	Neg
	Neg	Neg	Neg	Neg	Neg	Neg	Neg	Neg	8	1015	Neg
	Neg	Neg	Neg	Neg	Neg	Neg	Neg	Neg	6	1020	Neg
	Neg	Neg	Neg	Neg	Neg	Neg	Neg	Neg	7	1020	Neg
	Neg	Neg	Neg	Neg	Neg	Neg	Neg	Neg	6	1015	Neg
Control (male)	Neg	Neg	Neg	Trace	Neg	Neg	Neg	Neg	7	1010	Neg
	Trace	Neg	Neg	Neg	Neg	Neg	Neg	Neg	7	1015	Neg
	Neg	Neg	Neg	Neg	Neg	Neg	Neg	Neg	6	1020	Neg
	Neg	Neg	Neg	Neg	Neg	Neg	Neg	Neg	6	1020	Neg
	Neg	Neg	Neg	Neg	Neg	Neg	Neg	Neg	6	1020	Neg

*IV* intravenous; *SG* specific gravity; *Neg* negative; *Pos* positive

### Histopathological evaluations indicated normal state of the selected tissues

**Fig. 3:**
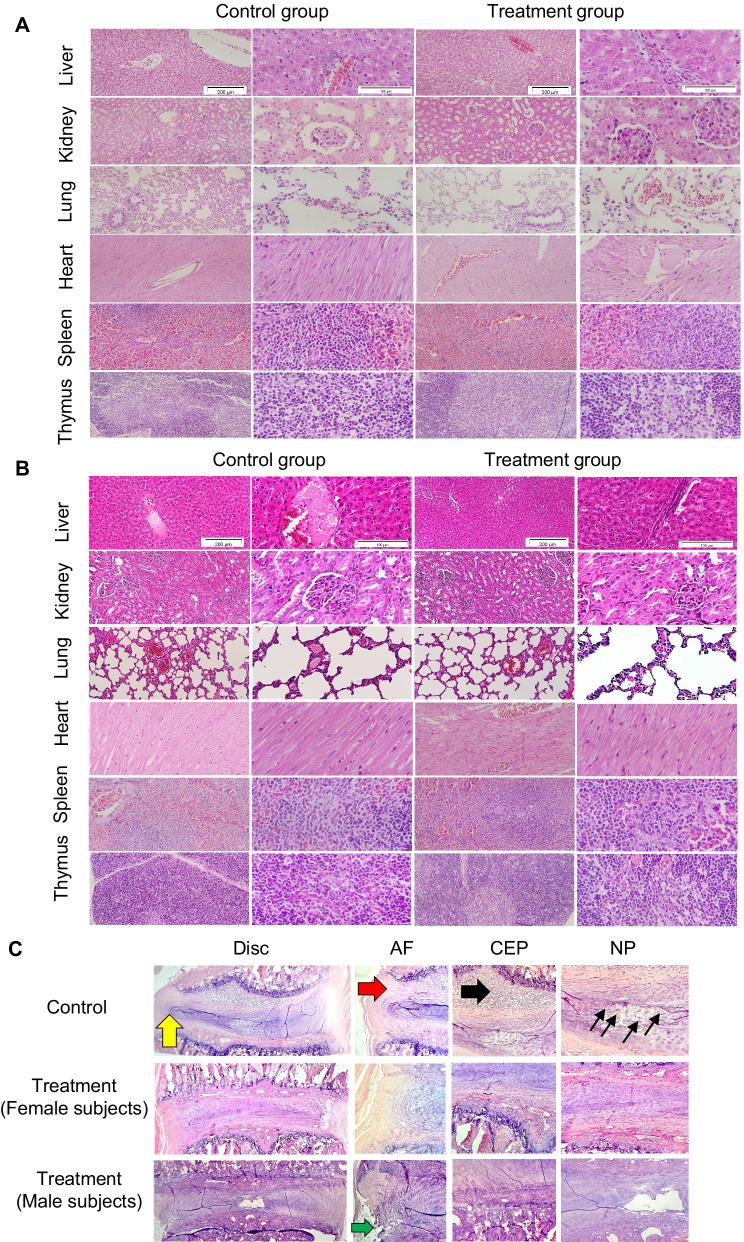
Histopathological analysis. H&E staining of the histopathological sections of the liver, heart, kidneys, spleen, thymus, and lungs in the experimental groups. Scale bars: 100x (200 μm), 400x (50 μm). **A** IVD groups, **B** IV groups, and **C** histopathological findings after IVD administration in the different experimental groups. In the control group, the degenerated disc exhibited a loss of normal height and central NP, disorganization of the lamellar arrangement of the annulus fibrosus, remodeling, and thickening of the cartilaginous endplate. Histopathological evaluation of the treatment group (female and male subjects) in both genders showed a loss of normal disc height in these groups. Yellow arrow: disc prolapse; red arrow: AF degeneration; black arrow: thickening and remodeling of the CEP; thin back arrows: NP cell necrosis; green arrow: needle entry site. H&E: hematoxylin and eosin; IVD: intervertebral disc; IV: intravenous, NP: nucleus pulposus; AF: annulus fibrosus; CEP: cartilaginous endplate

Figure 3 shows the representative images of the H&E-stained heart, kidney, liver, spleen, thymus, and lung tissues, and discs (IVD injection group). Typical normal structures of different tissues were visible in both the control and treatment groups. Histopathological evaluations did not show any changes such as inflammatory response or necrosis in the treatment groups (Fig. 3). Overall, all harvested tissues were normal, and the histopathological findings excluded the occurrence of any damage or toxicity.

### Nude mice did not show teratoma formation after BM-cMSC injection

No trace of teratoma formation was observed in the treatment and negative control groups during the three-month follow-up. However, tumors (1.2 ± 0.4 cm) were observed from days 14 to 18 in the positive control group (embryonic stem cell; data not shown).

## Discussion

Preclinical studies are critical for recognition of the potential risks and identification of possible side effects of cells that may occur in the clinical setting [33–35]; However, a “standard set” of preclinical tests and testing parameters that are uniformly applicable to all products does not exist [20].

The genetic and physiological differences between humans and animals present a challenging issue because animal findings cannot be fully generalized to humans [36, 37]. Therefore, preclinical studies are an initial step to determine the degree of safety in a living body.

In this study, we used the cells which had passed both quality control and safety tests in 12 to 15 passages through the process of establishing a related cell bank [16]. However, it is necessary to mention that the expanded ex vivo culture condition or long-term storage of cell therapy products may cause some alterations that affect both in vitro and in vivo results [16, 38]. Indeed, preclinical studies are needed to ensure the safety of the final BM-cMSC product, as a homogenous allogeneic product. Also, it should be noted that we used BM-cMSCs which were derived from a single young healthy donor. Although the establishment of a cell bank of BM-cMSCs by clonal selection could extremely decrease the need for a new BM donor [16], when a donor change occurs for whatever reason, all of the toxicity study evaluations should be performed again because of donor-to-donor variations.

Preclinical studies are often not very large. However, they must provide detailed information on dosing regimen, clinical route of administration, adequate study duration, standard toxicology assessment, and specific safety considerations [20].

In the present study, we investigated the standard toxicity and safety of BM-cMSCs developed under GMP-compatible conditions at Royan ATMP-TDC. We designed the present preclinical study in accordance with regulatory guidelines for cell therapy, medical devices, and medical equipment in GLP from the US Food and Drug Administration (US FDA) [39] and the European Medicines Agency (EMA) [21, 23, 24, 26], with some modifications based on available conditions [13–15, 17, 18]. Here, we conducted a standard toxicity evaluation (mortality, clinical observations, body weight, clinical pathology, and macro- and microscopic examinations) of BM-cMSCs administered via the IVD and IV injection routes within a defined period of time. The BM-cMSCs were injected as a single dose in both the systemic and localized routes in accordance with the intended clinical use, IV to treat systemic diseases such as RA [40] and local injection, and IVD for degenerated discs in patients with LBP [41]. These routes of administration were assessed in both sexes of rats.

Assessments were performed daily during the subacute period for 14 days and twice per week in the subchronic period for 90 days. After the rats were euthanized, we assessed hematological (injury to the hematopoietic system) and biochemical parameters (electrolyte balance, carbohydrate metabolism, and liver and kidney function), urinalyses (changes in normal excretory functions caused by biological agents), and histopathology of the rat tissues. As mentioned, these items are essential variables in the assessment of standard toxicity and safety of drug and biological agents. The experiments showed that BM-cMSCs had no measurable systemic toxicities at different clinical and preclinical levels after the systemic and local injections over the short term and long term compared to the control female and male rats.

In the subacute phase of the IVD group, both hemoglobin and hematocrit levels of the treatment and control groups were lower than the normal range. We attributed this to bleeding from surgery, which resolved in the chronic phase.

In a few cases of urinalysis, there was a weak reaction that was not further investigated due to its low value. This could be due to contamination of the tip of the syringe by abdominal fluid during aspiration of urine from the bladder.

Although cell efficiency was not considered in this study, histopathological evaluation of the IVD routes in the treatment group for both genders showed a reduction in the number of nucleus pulposus (NP) cells compared to normal disc; however, these numbers were considerably higher than those of the negative control (sham). Moreover, although many studies have shown that MSCs do not survive more than a few days after injection [42, 43], we did not use the methods for tracking the cells by labeling as they could cause interference in cell toxicity evaluations [44].

In term of cell tumorigenicity, although non-tumorigenicity of MSCs has been proven in human clinical studies of acute myocardial infarction, osteoporosis, and GvHD, there are some reports where MSCs can induce sarcomas or facilitate tumor growth [45]. Thus, in the present study, we assessed the tumorigenicity of cMSCs in nude mice; the results after 3 months showed no tumorigenicity by the injected cells.

Similar results were obtained by Choi et al. [46] where they sought to determine the safety of adipose tissue-derived MSCs (AT-MSCs). They reported that local injection of AT-MSCs had no adverse effects in terms of clinical symptoms, hematology and biochemistry parameters, urinalysis, weight, water and food consumption, and mortality and also, tumor formation was not observed. However, only local transplantation was performed at the femoral bone site and only male animals were selected in their study. Both sexes should be used for toxicity studies because of the physiological differences between males and females and also based on developing guidelines and recommendations [20].

In order to determine the toxicity and tumor formation of AT-MSCs, Ra et al. [47] administered different doses of AT-MSC by systemic injection into the tail veins of male and female SCID mice. They did not observe any side effects or tumor formation. They used different doses of cells (5 × 10^6^, 2.5 × 10^8^, and 3.5 × 10^7^ cells/kg body weight). Doses above 3.5 × 10^7^ cells/ kg body weight also had no visible toxicity effects during the 90-day study period. They also used the cells in a phase I clinical trial for transplantation into patients with spinal cord injuries. The patients who received a systemic injection of 4 × 10^8^ cells had no side effects during a 12-month follow-up [47, 48].

Another study by Beggs et al. [49] showed that systemic (IV) administration of allogeneic human BM-MSCs at a dose of 5 × 10^6^ cells/kg of body weight in monkeys followed by intramuscular injection of these cells at the same dose had no side effects on general health or immune response of these monkeys.

Our findings are consistent with a study conducted by Aithal et al. [50] who evaluated IV administration of low and high doses of human BM-MSCs in rats. There were no symptoms of abnormal body weight, decreased water and food consumption, behavioral changes, general clinical signs such as level of activity, posture and hair loss, and mortality observed during the 30-day period. Rengasamy et al. [5] assessed safety and toxicity of human BM-MSCs in studies of rodents and non-rodents. In their study, the BM-MSCs were obtained either from a single donor or a mixture of multiple donors and administration in single or repeated doses. The results showed that single or multiple doses of pooled BM-MSCs had no acute or subchronic systemic or local toxic effects in healthy animals.

Kannaiyan and colleagues [51] sought to determine the safety and acute cytotoxicity of Wharton jelly-derived MSCs. For this purpose, they assessed Swiss albino mice (from both sexes) after injecting MSCs at the dose of 10 × 10^6^ MSCs/kg body weight intravenously and subcutaneously. They demonstrated that injected MSCs did not induce any mortality, abnormal clinical signs, adverse effect on the body weights, and visible pathological changes on target tissues during the period of the study. Therefore, the authors concluded that the cells were safe for cell therapy purposes. However, they only assessed acute toxicity over 14 days.

Although we did not use the labeled cells for tracking them in different tissues such as pulmonary passage, which frequently reported as a major obstacle for intravenous stem cell delivery [52], no clinical signs of respiratory distress were observed in MSC-administered animals in this study. Also, the histopathological evaluations did not show any changes such as inflammatory response or necrosis in the treatment groups. It seems that the pulmonary embolism or cell trapping after infusing the cells intravenously depends on both the concentration and flow rate of cell delivery. The pulmonary emboli and increased mortality were found in IV-delivered animals at higher cell dose/flow rate [53–55]. In particular, concentrations above 1.0 × 10^7^ cells/ml by IV administration in mice lead to insufficient dilution of the cells in the blood, resulting in occlusion in the first capillary bed that is encountered (i.e., pulmonary embolism) and a significant increase in mortality rate. Regarding this point, in this study we had diluted 6 × 10^6^ cells/kg in 500 μl normal saline and used a slow delivery flow rate to avoid this frequent problem.

In general, a review of studies show a small number of articles published that pertain to cell-based products safety testing. It is necessary to conduct preclinical studies to determine the safety of MSCs, and this leads to a better understanding of the impact of cellular products on immune and physiological responses in the body and helps researchers to predict immune characteristics and their respective responses in humans. Although these studies are not entirely consistent with our study in terms of injectable cell doses, our source of MSCs, route of administration, and type of animal are in line with the results of similar studies. The results of the current study indicated that no signs of tumorigenicity and toxicity were observed after IVD or systemic administration of allogeneic or xenogeneic BM-cMSCs. This study was one of the preclinical study phases of BM-cMSCs before the application of these cells in the clinical trials. However, there is always a concern about the efficiency of cell therapy in clinical applications. For example, it is shown that the transplantation of cryopreserved human umbilical cord blood mononuclear cells had not efficient therapeutic effects in rat models of stroke [56]. Therefore, we are investigating the efficacy of the BM-cMSCs in comparison with other sources of MSCs in various animal disease models such as rheumatoid arthritis and low back pain (unpublished data). Indeed, both preclinical toxicity and efficacy of the BM-cMSCs in various disease models and specific safety considerations, such as immunological changes, need to be assessed before the clinical use of these cells.

## Conclusion

The results of this study demonstrated that a single dose of administration of xenogeneic BM-cMSCs did not cause systemic or localized toxic effects in both sexes of normal SD rats. These findings were shown by behavioral changes, clinical signs of toxicity, changes in body weight, water and food consumption, and histopathological findings. Few published studies have comprehensively investigated the toxicity of BM-cMSCs, so preclinical toxicity and efficacy of the BM-cMSCs need to be assessed in animal models before clinical use.

## Supplementary Information

**Additional file 1.** **Tables S1-S7** presenting behavioral and clinical symptoms, average body weight and food and water consumption of the subjects in different groups and stages of the study.

## Data Availability

All other data generated or analyzed during this study are included in this published article and its supplementary information files.

## Abbreviations

MSCs: Mesenchymal stromal cells
GMP: Good manufacturing practice
BM-cMSCs: Bone marrow-derived clonal MSCs
ISCT: International Society for Cell and Gene Therapy
SD: Sprague Dawley
IV: Intravenous
IVD: Intervertebral disc
cLBP: Chronic low back pain
GvHD: Graft-versus-host disease
OA: Osteoarthritis
RA: Rheumatoid arthritis
MS: Multiple sclerosis
ATMP: Advanced therapy medicinal product
Alpha MEM: Minimum Essential Medium Eagle-Alpha Modification
FBS: Fetal bovine serum
ICB: Initial cell bank
MCB: Master cell bank
WCB: Working cell bank
EoPCB: End of production cell bank
NBF: Neutral buffered formalin
H&E: Hematoxylin and eosin
hESC: Human embryonic stem cell
AT-MSC: Adipose tissue-derived MSCs
NP: Nucleus pulposus
AF: Annulus fibrosus
CEP: Cartilaginous endplate
