# Supplementary material for: Standard toxicity study of clinical-grade allogeneic human bone marrow-derived clonal mesenchymal stromal cells
Source: Stem Cell Res Ther. 2022 May 26;13:213. doi: 10.1186/s13287-022-02899-9 (PMC9137136; doi:10.1186/s13287-022-02899-9)
Supplement: Supplementary file 1 — Additional file 1. Tables S1-S7 presenting behavioral and clinical symptoms, average body weight and food and water consumption of the subjects in different groups and stages of the study. [file 13287_2022_2899_MOESM1_ESM.docx]

**Additional file 1:**

| **Table S1.** **Behavioral and clinical symptoms.** | | | | | | | | | | | |
| --- | --- | --- | --- | --- | --- | --- | --- | --- | --- | --- | --- |
| **Subacute phase** | **D1** | **D2** | **D3** | **D4** | **D5** | **D6** | **D7** | **D8** | **D9** | **D…** | **D15** |
| **Chronic phase** | **W3** | **W4** | **W5** | **W6** | **W7** | **W8** | **W9** | **W10** | **W11** | **W12** | **W13** |
| **Body weight (g)** |  |  |  |  |  |  |  |  |  |  |  |
| **Morbidity** |  |  |  |  |  |  |  |  |  |  |  |
| **Level of activity** |  |  |  |  |  |  |  |  |  |  |  |
| **Posture** |  |  |  |  |  |  |  |  |  |  |  |
| **Respiration** |  |  |  |  |  |  |  |  |  |  |  |
| **Hydration status** |  |  |  |  |  |  |  |  |  |  |  |
| **Stereotypic behavior** |  |  |  |  |  |  |  |  |  |  |  |
| **Ears** |  |  |  |  |  |  |  |  |  |  |  |
| **Nose** |  |  |  |  |  |  |  |  |  |  |  |
| **Oral cavity** |  |  |  |  |  |  |  |  |  |  |  |
| **Enlarged abdomen** |  |  |  |  |  |  |  |  |  |  |  |
| **Eye lesion/bulging** |  |  |  |  |  |  |  |  |  |  |  |
| **Facial swelling** |  |  |  |  |  |  |  |  |  |  |  |
| **Hair loss** |  |  |  |  |  |  |  |  |  |  |  |
| **Itching/scabs** |  |  |  |  |  |  |  |  |  |  |  |
| **Sneezing** |  |  |  |  |  |  |  |  |  |  |  |
| **Limbs, feet, tail** |  |  |  |  |  |  |  |  |  |  |  |
| **Urinary discharge** |  |  |  |  |  |  |  |  |  |  |  |
| **Vaginal/testicular issues** |  |  |  |  |  |  |  |  |  |  |  |
| **Reactivity to handling** |  |  |  |  |  |  |  |  |  |  |  |
| **Palpation of masses** |  |  |  |  |  |  |  |  |  |  |  |
| D: Day; W: Week | | | | | | | | | | | |

| **Table S2. Average body weight and percentage of body weight gain on day one of the experiment and days 14 (subacute) and 90 (subchronic) after injection of hBM-cMSCs in the IVD groups.** | | | | | |
| --- | --- | --- | --- | --- | --- |
| **Group** | | **Animals (n)** | **Mean ± SD (day0)** | **Mean ± SD** | **Body growth rate (%)** |
| **Subacute (day 14)** | **Treatment** | 11 | 250.34 ± 39.67 | 270.37 ± 45.66 | 7.4 |
|  | **Control** | 12 | 258.67 ± 50.13 | 277.37 ± 54.18 | 6.7 |
| **Subchronic (day 90)** | **Treatment** | 12 | 266.20 ± 48.21 | 331.77 ± 75.81 | 19.7 |
|  | **Control** | 12 | 268.12 ± 46.76 | 337.84 ± 72.45 | 20.6 |
| BM-cMSC: Bone marrow-derived clonal mesenchymal stromal cells; IVD: Intervertebral disc | | | | | |

| **Table S3. Average body weight and percentage of body weight gain on the day one of the experiment and days 14 (subacute) and 90 (subchronic) after injection of hBM-cMSCs in the IV groups.** | | | | | |
| --- | --- | --- | --- | --- | --- |
| **Group** | | **Animals (n)** | **Mean ± SD (day0)** | **Mean ± SD** | **Body growth rate (%)** |
| **Subacute (day 14)** | **Treatment** | 12 | 264.99 ± 52.67 | 289.23 ± 61.90 | 8.3 |
|  | **Control** | 12 | 263.55 ± 47.31 | 285.87 ± 52.04 | 7.8 |
| **Subchronic (day 90)** | **Treatment** | 12 | 259.92 ± 50.11 | 324.68 ± 80.92 | 19.9 |
|  | **Control** | 11 | 264.89 ± 50.14 | 333.10 ± 73.33 | 20.4 |
| BM-cMSC: Bone marrow-derived clonal mesenchymal stromal cells; IV: Intravenous | | | | | |

| **Table S4.Water consumption in the IVD groups during the study of subacute and subchronic toxicities.** | | | | |
| --- | --- | --- | --- | --- |
| **Group** | | **Animals (n)** | **Water consumption (ml)** | **P-value** |
| **Subacute** | **Treatment** | 11 | 45.16 ± 3.86 | 0.3595 |
|  | **Control** | 12 | 46.41 ± 2.41 |  |
| **Subchronic** | **Treatment** | 12 | 34.27 ± 2.79 | 0.6666 |
|  | **Control** | 12 | 34.82 ± 3.39 |  |
| P≤.05 indicates statistical significance. IVD: Intervertebral disc | | | | |

| **Table S5.** **Water consumption in the IV groups during the study of subacute and subchronic toxicities.** | | | | |
| --- | --- | --- | --- | --- |
| **Group** | | **Animals (n)** | **Water consumption (ml)** | **P-value** |
| **Subacute** | **Treatment** | 12 | 36.30 ± 2.02 | 0.5549 |
|  | **Control** | 12 | 36.00 ± .52 |  |
| **Subchronic** | **Treatment** | 12 | 32.94 ± 3.82 | 0.2394 |
|  | **Control** | 11 | 34.64 ± 2.79 |  |
| P≤.05 indicates statistical significance. IV: Intravenous | | | | |

| **Table S6. Average daily (subacute) and weekly (subchronic) food consumption in the IVD groups.** | | | | |
| --- | --- | --- | --- | --- |
| **Group** | | **Number of animals** | **Food consumption (g)** | **P-value** |
| **Subacute** | **Treatment** | 11 | 19.15 ± 1.31 | 0.7869 |
|  | **Control** | 12 | 18.98 ± 1.63 |  |
| **Subchronic** | **Treatment** | 12 | 18.62 ± 1.11 | 0.0544 |
|  | **Control** | 12 | 17.63 ± 1.2 |  |
| P≤.05 indicates statistical significance. IVD: Intervertebral disc | | | | |

| **Table S7.Average daily (subacute) and weekly (subchronic) food consumption in the IV groups.** | | | | |
| --- | --- | --- | --- | --- |
| **Group** | | **Number of animals** | **Food consumption (g)** | **P-value** |
| **Subacute** | **Treatment** | 12 | 16.63.45 ± 2.54 | 0.2064 |
|  | **Control** | 12 | 15.47 ± 0.50 |  |
| **Subchronic** | **Treatment** | 12 | 18.14 ± 2.49 | 0.9819 |
|  | **Control** | 11 | 18.16 ± 1.07 |  |
| P≤.05 indicates statistical significance. IV: Intravenous | | | | |
